# Simultaneous determination of lesinurad and co-administered drugs used in management of gout comorbidities to uncover potential pharmacokinetic interaction in rat plasma

**DOI:** 10.1038/s41598-025-93680-4

**Published:** 2025-04-02

**Authors:** Hadeel A. Khalil, Amira F. El-Yazbi, Eman I. El-Kimary, Mohamed A. Elrewiny, Ahmed F. El-Yazbi, Tarek S. Belal

**Affiliations:** 1 Pharmaceutical Analytical Chemistry Department, Faculty of Pharmacy, Alexandria University, Alexandria, 21521 Egypt; 2 Faculty of Pharmacy and the Research and Innovation Hub, Alamein International University, Alamein, 51718 Egypt; 3 Department of Pharmacology and Toxicology, Faculty of Pharmacy, Alexandria University, Alexandria, 21521 Egypt

**Keywords:** Etoricoxib, Gout, HPLC, Lesinurad, Pharmacokinetics, Therapeutic drug monitoring, Analytical chemistry, Bioanalytical chemistry

## Abstract

Gout is one of the most prevalent forms of arthritis that is usually accompanied by other comorbidities. For this reason, multiple drugs are routinely prescribed for gout patients, which may affect the clinical course outcomes, and increase the risk of drug-drug interactions. This work presents a novel, simple, and sensitive high performance liquid chromatography (HPLC) method for the simultaneous determination of lesinurad (LES) and other co-administered drugs that are subject to potential pharmacokinetic interactions such as etoricoxib (ETC), eplerenone (EPL), and amiodarone (AMD) in rat plasma. Moreover, a pharmacokinetic study was conducted by co-administration of LES and ETC to rats to assess any possible alteration in their pharmacokinetic profiles and the obtained samples were analyzed using the developed method. Chromatographic separation was achieved using a gradient elution of a mobile phase consisting of acetonitrile and potassium dihydrogen orthophosphate buffer, pH 4.2 on a Zorbax Eclipse Plus C18 (4.6 × 250 mm, 5 μm particle size) column. The developed method exhibits adequate sensitivity with a LLOQ of 100 ng/mL and was successfully validated as per the FDA bioanalytical guidelines and was found to be linear over the range from 100 to 50,000 ng/mL for all the selected drugs. The results of the pharmacokinetic study showed an increase in the area under the curve (AUC) of each of the two drugs (LES and ETC) following the repeated administration of the other. This raises concerns of the possible renal injurious effect of ETC when co-prescribed with LES. Moreover, this work uncovers the necessity for therapeutic dose adjustment or increased clinical vigilance for side effects and/or lack of efficacy upon concomitant administration of the selected drugs to gout patients.

**Supplementary Information:**

The online version contains supplementary material available at 10.1038/s41598-025-93680-4.

## Introduction

Gout is one of the most common forms of arthritis. It is associated with the deposition of monosodium urate crystals resulting from altered purine metabolism caused by hyperuricemia^1,2^. Gout incidence is more prevalent among the elderly, especially postmenopausal women. According to the data from the National Health and Nutrition Examination Survey (NHANES), gout nearly affects 83 million adults in the USA, about 3.9% of the whole population^3^. Additionally, recent epidemiologic studies suggests a rising incidence of gout in the next few years due to the increasing prevalence of obesity and dyslipidemia around the world^4,5^.

From a clinical perspective, gout and/or hyperuricemia are typically associated with several medical conditions, including obesity, dyslipidemia, diabetes mellitus, hypertension, cardiovascular disorders, and most importantly renal impairment^6^. This necessitates multiple prescription drug use, which may influence the clinical course of gout, and increases the risk of drug-drug interactions. Indeed, drug-drug interactions (DDIs) are one of the key causes of drug-related problems in developed countries, especially due to poly-therapy. Unfortunately, DDIs that primarily cause a change in the pharmacokinetics (PK) of a given medication will produce a consequent alteration in its pharmacodynamics (PD), thereby possibly harming the patient due to the development of adverse drug reactions, reduced clinical efficacy, or potentially leading to toxicity^7^. As such, in order to avoid possible DDIs, gout patients may require treatment of their own accord and some modifications for gout management^6,8^.

**Fig. 1:**
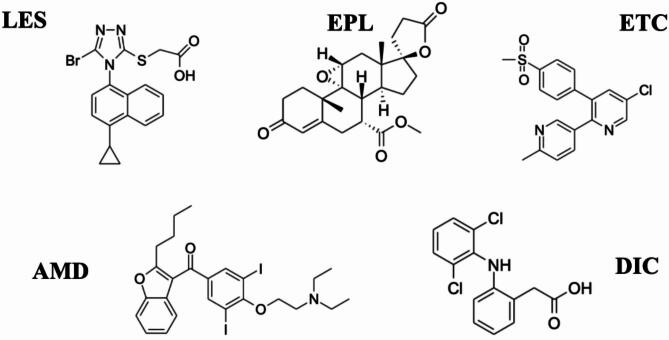
Chemical structures of the investigated drugs lesinurad (LES), eplerenone (EPL), etoricoxib (ETC), amiodarone (AMD) and the internal standard diclofenac (DIC).

In this context, lesinurad (LES) (Fig. 1) is an FDA approved selective uric acid reabsorption inhibitor (SURI) used in the treatment of hyperuricemia associated with gout in combination with xanthine oxidase inhibitors (XOIs)^9,10^. While xanthine oxidase inhibitors primarily reduce uric acid synthesis in the liver, LES reduces serum urate levels by inducing a uricosuric effect through the reduction of renal uric acid reabsorption via the inhibition of the uric acid transporter URAT1^11^. Of note, a proclaimed advantage of LES is that it was devoid of the potential off-target and adverse effects associated with other URAT1 inhibitors^11^. Nevertheless, significant clinical concern remained regarding the potential nephrotoxic effects of LES^12^, especially in light of the observation that its inhibitory effect extends to other transporters, including the organic anion transporter protein, OATP1B1, organic cation transporter, OCT1, and the organic anion transporter, OAT3 and OAT4 ^13^, with its effects on OAT4 occurring at clinically relevant doses^11^.

Of note, non-steroidal anti-inflammatory drugs (NSAIDS) are commonly used to treat the pain and inflammation associated with gout, particularly in the acute phase^13^. Importantly, evidence has shown that selective inhibitors of cyclo-oxygenase-2, such as etoricoxib (ETC), were associated with fewer side effects in gout patients, and less therapy withdrawal^13^. However, ETC, while superior to other NSAIDs in having significantly less gastric toxicity with equal efficacy, still retains considerable renal toxicity similar to other NSAIDs^14^. Indeed, ETC does not interfere with the function of hepatic cytochrome P450 (CYP) enzymes, and hence does not pose a meaningful clinical risk due to metabolic DDIs^14^. Nevertheless, ETC is a substrate and inhibitor of the organic anion transporter^15^, possibly altering the pharmacokinetic and pharmacodynamic profile of LES. Under such circumstances, the outcome of the said interactions might bear significant detrimental consequences for gout patients with co-morbid renal and cardiovascular disease. Relatedly, similar interactions were of concern with LES, and previous literature examined such possibilities for drugs such as metformin and atorvastatin^16^. Yet to our knowledge, no similar studies examined this for ETC, being a drug of a high likelihood of co-administration. In this regard, the development of a method that can quantitate ETC, as well as LES, in the biological setting of measuring the alteration of PK profile of either drug is of paramount importance. Significantly, such a method will serve as a *Universal tool* in the investigation of other potential interactions of LES, being also a CYP3A4 inducer^17^, with medications co-administered to address cardiovascular comorbidities. Indeed, many such medications are CYP3A4 substrates, including eplerenone (EPL) and amiodarone (AMD)^18^.

Over the past years, literature covered several techniques for the analysis of LES in bulk and in pharmaceutical dosage forms including an HPLC-stability indicating study on LES alone^19^. Simultaneous determination of LES and allopurinol (ALP) was performed using reversed phase HPLC^20,21^, Capillary Zone Electrophoresis (CZE)^22^, spectrophotometric^23–25^, and spectrofluorimetric methods^26^. On the other hand, several techniques were developed for the analysis of LES in biological fluids. Ultra-performance HILIC with tandem mass spectrometry was used for the quantitation of LES in rat plasma^27^. Moreover, a spectrofluorimetric method was applied for LES and ALP determination in human plasma^28^. Another method was developed for LES and febuxostat (FEB) determination using micelle-enhanced conventional and synchronous spectrofluorimetry^29^. Moreover, a green reversed-phase HPLC method with fluorescence detection for the simultaneous determination of LES, FEB, and diflunisal was established^30^. To the best of our knowledge, there were no reports for the simultaneous determination of LES, EPL, ETC, and AMD in rat plasma.

In the present study, we developed and optimized a novel, simple, and sensitive High Performance Liquid Chromatography (HPLC) method for the determination of LES and other co-administered drugs (ETC, EPL, AMD) that are subject to potential pharmacokinetic interactions in plasma. A NSAID, diclofenac (DIC), which is commonly used in acute pain attacks associated with gout has been used as an internal standard. The developed analytical method is capable of measuring more than one drug present in low concentrations in different biological matrices and was used to facilitate the in vivo assessment of pharmacokinetic alterations due to the interaction between LES and ETC in rats.

## Experimental

### Materials and reagents

LES powder (purity > 99.85%) was purchased from Sigma-Aldrich Chemie GmbH (Ontario, Canada). AMD (purity > 99.80%), and DIC (purity > 99.90%) powders were provided by Pharco Pharmaceuticals, Co. (Alexandria, Egypt). While ETC and EPL powders (purity > 99.85%) were a kind gift from Mash Premiere Pharmaceuticals, Co. (Cairo, Egypt) and SAJA Pharmaceuticals, Co. (6th of October, Egypt) respectively. Zurampic tablets containing 200 mg of LES per tablet (B.No. 1275, manufactured by Astrazeneca) was generously supplied by the National Organization for Drug Control and Research, Giza, Egypt. Arcoxia tablets labelled to contain 120 mg of ETC per tablet (B.No. 1258, manufactured by Frosst Iberica SA via Complutense 140, Alcala De Henares, Madrid, E-28805, Spain) was purchased from the local market. HPLC grade Methanol and acetonitrile (Fisher Scientific UK Limited, Loughborough, Leicestershire, UK), analytical grade potassium dihydrogen orthophosphate (Riedel-de-Haën, Germany) and high purity deionized water were used.

### Instrumentation and chromatographic conditions

The HPLC-DAD system consisted of Agilent 1200 series (auto-injector, quaternary pump, vacuum degasser and diode array and multiple wavelength detector G1315 C/D and G1365 C/D) connected to a computer loaded with Agilent ChemStation Software (Agilent Technologies, Santa Clara, CA, USA). The DAD wavelength was set at 225 nm for LES, 242 nm for EPL and AMD, and 280 nm for the detection of ETC and DIC (IS). The chromatographic separation was achieved using a Zorbax Eclipse Plus C18 (4.6 × 250 mm, 5 μm particle size) column attached to Zorbax Eclipse Plus C18 (4.6 × 12.5 mm, 5 μm particle size) analytical guard column (Agilent Technologies, Santa Clara, CA, USA). The gradient elution was composed of the mobile phase: acetonitrile and 0.05 M potassium dihydrogen orthophosphate eluted in the ratios and at flow rates listed in Table 1 using an injection volume of 50 µL. All determinations were performed at 25º C. All pH measurements were made using a Hanna pH meter, (RI, USA). Calibration was done using standard buffers of pH 4 and 7 at room temperature.

**Table 1:** Gradient elution of mobile phase applied in the chromatographic separation of using the proposed HPLC–DAD method.

Time (min)	Mobile phase composition		Flow rate (mL/min)
	Acetonitrile (%)	KH_2_PO_4_ Buffer (%)	
0–3	0	100	1
3.1–6	40	60	1
6.1–7	50	50	1
7.1–12.5	70	30	1.5

### Stock and standard solutions

Stock solutions of LES, ETC, EPL, AMD (1000 µg/mL) and the internal standard (DIC) were separately prepared in methanol. To prepare samples for the calibration curves and validation assessment, three working standard solutions (100, 10 and 1 µg/mL) of all the drugs were prepared freshly by successive 1/10 dilutions of the stock solutions with methanol. In addition, a working standard solution of DIC (100 µg/ml) was also freshly prepared in in methanol.

### Extraction procedure

The internal standard DIC (0.05 mL) was added to each 0.2 mL rat plasma sample. To the rat plasma sample, 0.6 mL of acetonitrile were added. In order to equalize the final volumes in both; the standard calibration curve samples and the rat plasma samples, up to 1 mL of methanol were added. The samples were covered, vortexed for 1 min at high speed followed by centrifugation for 10 min at ~ 2500 × g. The organic layer was transferred to HPLC autosampler vials. For each sample, 50 µL were directly injected into the HPLC system.

### Recovery

The plasma recoveries were determined for LES, ETC, EPL, AMD and DIC at concentration level 2500 ng/mL in rat plasma using four replicates for each concentration. The extraction efficiency was estimated by comparing the obtained peak areas of each analyte to those of unextracted standards.

### Calibration curves and quality control samples

Calibration curves were constructed using samples of 0.2 mL rat plasma containing LES, ETC, EPL, AMD and DIC (IS). The curve ranged from 100 to 50,000 ng/mL for the selected drugs. For each individual drug, the ratio of its peak area to IS peak area was calculated and plotted versus the expected drug concentration. Owing to the wide range of concentrations, the calibration curve data were weighed by a factor of 1/x^2^ for all drugs.

For preparation of the quality control samples, drug-free plasma samples were spiked with appropriate aliquots of LES, ETC, EPL, and AMD working standard solutions, and prepared in duplicates. The concentrations were 100, 200, 5000, 25,000 ng/ml for all the studied drugs. DIC concentration, in all samples, was 250 ng/ml.

### Validation

The US FDA guidelines for bioanalytical method validation were followed for method validation^31,32^.

#### Selectivity

Matrix interference was tested by comparing six blank plasma samples with those spiked with the analytes at their LLOQ concentration levels.

#### Accuracy, precision and recovery

Intraday accuracy and precision of the assay were determined using four sample replicates of 100, 1000, 10,000, and 25,000 ng /mL for the entire set of drugs in rat plasma on the same day. For estimation of the inter-day accuracy and precision, the same concentrations were analyzed in three separate days. For each daily run, concentrations were determined by comparison with a calibration curve prepared simultaneously on the same day of analysis. Percentage coefficient of variation (CV%) was used to assess precision while bias was assessed using percentage error of the mean.

#### Linearity, range and sensitivity

Calibration graphs were constructed by preparation of blank sample, a sample fortified with IS (zero sample), in addition to nine plasma samples fortified with the selected drugs (in addition to IS) within concentration ranges listed in Table 2, including their LLOQs. After injecting the samples, the calibration graph for each drug was obtained by plotting the peak area ratios of each drug and the IS against the corresponding drug concentration.

**Table 2:** Regression and statistical parameters for the proposed HPLC–DAD method.in rat plasma.-DAD method and statistical parameters for the proposatistical parameters for the proposed HPLC–DAD method.

Parameters	LES	EPL	ETC	AMD
Linearity range (ng*/*ml)	100–50,000	100–50,000	100–50,000	100–50,000
Intercept^a^	0.005	0.100	0.0186	0.0008
Slope^b^	0.0001	4.115	5.13 × 10^‒^	5.84 × 10^‒5^
Correlation coefficient (r)	0.9997	0.9996	0.9992	0.9993
^a^Sa	0.021	0.020	0.011	0.012
^b^S_b_	9.16 × 10^‒7^	8.83 × 10^‒7^	5.03 × 10^‒7^	5.43 × 10^‒7^
^c^Sy*/*x	0.049	0.048	0.027	0.029
S_b_%	0.574	2.16	0.981	0.930
F	30,347	2171	10,380	11,550
Significance F	1.32 × 10^‒15^	4.97 × 10^‒11^	9.62 × 10^‒14^	6.27 × 10^‒14^

^a^Sa is standard deviation of intercept,

^b^Sb is standard deviation of slope,

^c^Sy*/*x is standard deviation of residuals.

*LES* lesinurad, *EPL* eplerenone, ETC etoricoxib, *AMD* amiodarone.

#### Stability

Stability studies were conducted by preparing low, medium and high plasma QC samples in triplicates under different conditions and comparing them with freshly prepared quality control samples. Namely, three freeze-thaw cycles (freeze at -20 °C then thaw at room temperature for three cycles), bench-top stability testing for 6 h at room temperature and long-term stability testing for 35 days at -20 °C. Moreover, investigation of the stability of LES, ETC, EPL, AMD and DIC stock solutions was performed by keeping the stock solutions for 30 days at 4 °C or for 6 h at room temperature.

### Application to a drug interaction pharmacokinetic study

The animal study has been designed and the results have been reported in accordance with the ARRIVE Guidelines. All animal experiments were conducted according to an experimental protocol approved by the Institutional Animal Care and Use Committee at Alexandria University (Serial Number 135) and in compliance with the Guide for the Care and Use of Laboratory Animals of the Institute for Laboratory Animal Research of the National Academy of Sciences, U.S.A. Animals were obtained from the animal facility at the Research and Innovation Hub at Alamein International University. Single dose pharmacokinetics of LES and ETC, as well as the effect of repeated administration of one on the PK parameters of the other reflecting the PK interactions if any, were assessed as described in our previous work^33^. Since the parameters of the oral pharmacokinetic investigation in experimental animals, in terms of feeding and fasting durations, oral dosing timing and volumes, blood sampling, as well as time intervals of redosing, can be controlled with relative ease, rat group sizes of 5–6 were sufficient for the detection of statistical difference in previous studies carried out in our laboratory^34,35^, and in other groups^36,37^, including studies that looked into the pharmacokinetics of ETC and LES^27,38^. As will be seen below, the experiment was designed for paired comparison with before and ater measurements from the same rats, further increasing the power for statistical analysis. As such, ten healthy adult male Sprague-Dawley rats (8 weeks of age, ~ 250 g weight) were randomized into two groups of five rats each: LES PK group and ETC PK group. No criteria were set for animal allocation per group. Rats were housed in polycarbonate cages on wood shavings bedding at the animal facility at Alamein International University under controlled temperature (23 ºC) and humidity (45%) and a 12-hour dark/light cycle. Rats were given *ad libitum* access to drinking water and commercial rodent chow (21% protein, Al-Fajr Animal Feed, Beheira, Egypt) prior to and during the experiment. The PK parameters of single doses of each drug were determined at baseline in the respective group and once more after seven days of administration of the other drug, without a washout period. To elaborate, on Day 0, following twelve hours of fasting, rats in each group received a single dose of the respective drug by oral gavage (15 mg/Kg for LES^27^, and 5 mg/Kg for ETC^39^). Blood samples were withdrawn from the tail vein at 0, 0.5, 1, 2, 4, 8, 12, 24 and 48 h time points into EDTA tubes. Plasma was separated by five-minute centrifugation at 4 °C at 5,000 rpm, labelled and immediately stored at -20 °C until the time of analysis. To assess the impact of each one of the two drugs on the other, the LES PK group received daily oral doses of ETC (5 mg/Kg) for one week, and vice versa. On Day 7, following twelve hours of fasting, rats in each of the groups received a single dose of the respective drug and blood samples were once again withdrawn at the same time intervals. At the end of the experiment, rats were euthanized by decapitation and exsanguination after isoflurane anesthesia. Drug concentrations in the plasma were measured in the samples withdrawn at Day 0 and Day 7, and were used to construct plasma concentration vs. time curves. Pharmacokinetic parameters including the area under curve (AUC) and maximal plasma concentration (Cmax) were determined from the best fit values after non-linear regression using GraphPad Prism version 8 (GraphPad Software, La Jolla, CA), while the elimination constant (Ke) and half-life (t1/2) were determined from best fit values of one phase decay using plasma concentrations ranging from Cmax to 48 h. On the other hand, the absorption rate constant (Ka) was estimated using the method of residuals based on the single oral dose pharmacokinetic data^40^. Statistical comparisons were done using two-way repeated measures ANOVA or paired t-test as indicated in the legend Fig. 3. A P-value of < 0.05 was considered significant.

## Results and discussion

### Method development

#### Optimization of the chromatographic conditions

To obtain adequate separation between the studied drugs, the internal standard (IS), and endogenous matrix components, several chromatographic conditions were attentively investigated. Various trials were conducted to choose a stationary phase, mobile phase, IS and the detector wavelength parameters that provides optimum results.

Selection of the optimum column was reached after several trials on different column types. These included Agilent Zorbax (SB-C18, 4.6 × 250 mm, 5 µm), Agilent Zorbax (Eclipse plus C18, 4.6 × 150 mm, 3.5 µm) and Waters Symmetry ( C18, 3.9 × 150 mm, 5 µm). The best results were achieved by using Zorbax Eclipse Plus C18 (4.6 × 250 mm, 5 µm particle size) column regarding peak shape giving an asymmetry factor (Af)around 0.9 for the studied drugs and IS peaks in addition to reasonable run time with K’ values between 2.47 and 3.84. To protect the column from plasma contaminants and highly absorptive components, a Zorbax Eclipse Plus C18 guard column (4.6 × 12.5 mm, 5 μm particle size) was used.

Different aqueous systems were tested, including acetic, formic acids and phosphate buffer. Broader peaks with high-capacity factor values were obtained using acetic and formic acids. On the other hand, phosphate buffer has resulted in sharper symmetrical peaks with N between 50,090 and 143,299 and consequently better resolution (> 1.5) was obtained. Hence, phosphate buffer was chosen and optimum separation was noticed in the pH range 4–4.4. Eventually the buffer solution was adjusted at pH 4.2 for it has produced the optimum separation.

As for the organic modifier, methanol and acetonitrile in different compositions were studied. However, methanol has resulted in poor separation between LES and ETC and hence was disregarded. Using acetonitrile, better results were achieved regarding resolution (> 1.5) and the conveniency of the run time (K` in the range of 2.47–3.84), in the ratios listed in Table 3. Gradient elution of the mobile phase were tried in different ratios to achieve maximum separation between the eluted peaks Table 1. Eventually, the aqueous phosphate buffer was exclusively pumped for the first 3 min in the run to avoid any potential overlap of the drugs` peaks with the plasma proteins peaks that normally appear at the beginning of the run. This was followed by an increase in the proportion of the organic phase and the flow rate as illustrated in Table 1 to ensure optimum separation of the eluted drugs in a reasonable total run time.

**Table 3:** System suitability parameters for the proposed HPLC–DAD method.

Analyte	Rt (min)*	Capacity factor (K′)*	Selectivity (α)*	Resolution (R_S_)*	Asymmetry factor (A_f_) (symmetry)*	Theoretical plates, N (plates*/*m)*	Injection precision^†^
LES	8.69	2.47	–	–	0.99	90,042	1.82
EPL	9.27	2.71	1.05	3.5	0.93	141,412	1.24
ETC	9.62	2.84	1.04	3.0	0.93	143,299	1.36
AMD	10.6	3.24	1.08	5.2	0.88	60,940	0.69
DIC (IS)	12.1	3.84	1.03	1.5	1.19	50,090	0.96

^†^Calculated as RSD% of five determinations.

*Recommendations by US FDA guidelines (31): K′ 2–10, R_S_ ≥ 1.5, N > 2000, α ≥ 1.1, A_f_ 0.9–1.2 and injection precision† ≤ 2.

Several candidates were tested to be employed as an internal standard including ambroxol, bromhexine, celecoxib and diclofenac (DIC). The good separation from the studied drugs, plasma components and the remarkable extraction recovery (95.66 ± 1.87%) made DIC the best candidate. Maximum sensitivity and yield was attained by extraction of the chromatograms at the λ_max_ of each analyte using DAD i.e.; 225 nm for LES, 241 nm for EPL, AMD and 280 nm for ETC and DIC.

#### Optimization of the extraction method

**Fig. 2:**
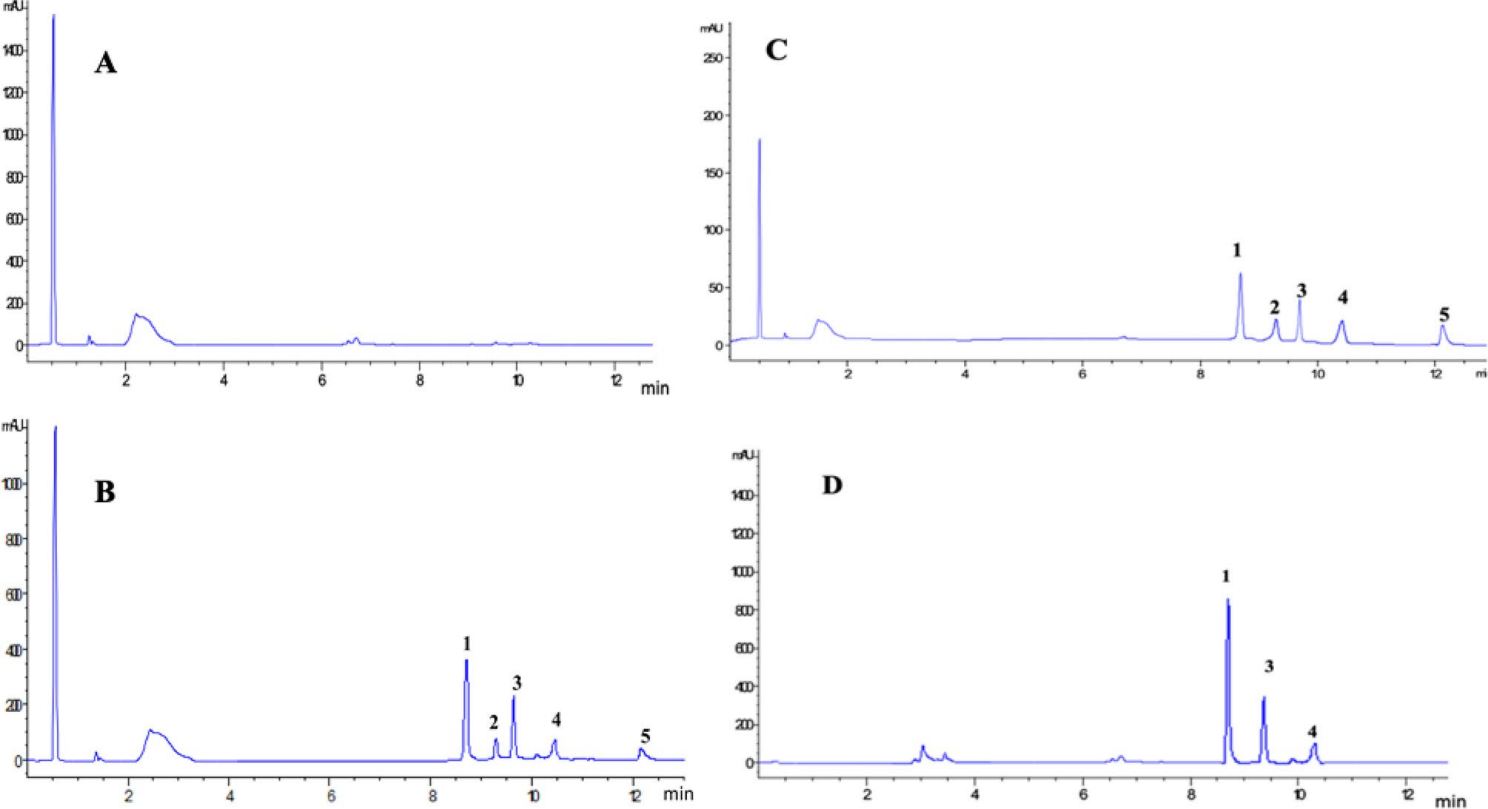
Representative HPLC chromatograms of (**A**) Blank plasma sample; (**B**) blank plasma spiked with 5 μg/mL LES (peak 1), 1 μg/mL ETC (peak 2), 1 μg/mL EPL (peak 3), 1 μg/mL AMD (peak 5) and the IS DIC (peak 4); (**C**) blank plasma spiked with the investigated drugs at their LLOQ; and (**D**) plasma sample obtained from a rat after 1 h of oral administration of 15 mg/Kg for LES, and 5 mg/Kg for ETC. Y-axis; mAU = Milli-absorbance unit. X-axis; min = time in minutes.

Liquid– liquid extraction and protein precipitation were studied to select an extraction method that provides maximum extraction recoveries of the investigated drugs from rat plasma. Liquid–liquid extraction using diethyl ether as an extracting solvent produced a noisy baseline as well as low percentage recoveries (nearly 60%). To overcome this problem, diethyl ether was substituted by ethyl acetate, despite the better percentage recoveries obtained (90%), a noisy baseline and the AMD peak was absent in all the obtained chromatograms. On the contrary, protein precipitation using acetonitrile showed higher percentage recoveries of the studied drugs (approximately 92%) and a consistent baseline and thus was selected as the optimum extraction method. Optimizing the chromatographic conditions and the extraction method has led to excellent resolution between the studied drugs and IS, and separation from the endogenous plasma components within 12 min (Fig. 2A,B).

### Validation

#### Selectivity

Chromatograms of blank rat plasma and blank rat plasma spiked with the investigated drugs at their LLOQs and DIC (IS) are displayed in Fig. 2. No interfering peaks were observed at the retention times of the studied drugs nor the IS.

Furthermore, the purity of the chromatographic peaks of the studied drugs recorded by DAD was checked to confirm selectivity. Peak purity was confirmed by plotting the similarity curve using all the spectra obtained during the elution of each peak (Supplementary Fig. 1). For each peak of the investigated drugs, the calculated purity factor lied below the purity threshold limit indicating peak purity of the studied drugs. The great accountability of the developed method was supported by the peak purity test results which showed no interference from endogenous plasma peaks.

#### Accuracy and precision

As per the FDA bioanalytical guidelines, the RSD% and Er% were (< 20%) at LLOQ and (< 15%) at the remaining concentrations. Hence, the validation data presented in Table 4 and (Table 1 in supplementary file); supports the good sensitivity, accuracy and precision exhibited by the proposed method. Furthermore, the mean obtained recoveries from rat plasma were in the ranges (86-118.9%). Reliable determination of the studied drugs in rat plasma was assured by the homogenous and reproducible recovery values obtained using this method.

**Table 4:** Intra-day and inter-day precision and accuracy for the determination of LES and ETC in rat plasma using the proposed HPLC–DAD method.

Selected Drug	Nominal concentration (ng/mL)	Mean % Recovery ± SD^a^ (ng/mL)	RSD%^b^	E_r_%^c^
LES				
Within-day	100	101.5 ± 12.9	12.7	1.53
	1000	103.5 ± 14.4	13.9	3.56
	10,000	97.50 ± 7.29	7.48	2.53
	25,000	107.6 ± 10.1	9.38	7.60
Between-day	100	110.7 ± 9.31	8.41	10.7
	1000	111.5 ± 6.91	6.20	11.5
	10,000	96.19 ± 3.52	3.66	3.81
	25,000	94.11 ± 11.9	12.6	5.89
ETC				
Within-day	100	102.7 ± 18.6	18.1	2.72
	1000	86.00 ± 6.27	7.29	14.0
	10,000	88.01 ± 8.04	9.14	12.0
	25,000	103.2 ± 6.32	6.12	3.22
Between-day	100	101.5 ± 6.32	6.22	1.50
	1000	102.4 ± 14.3	14.0	2.40
	10,000	96.6 ± 13.3	13.8	3.40
	25,000	95.7 ± 6.58	6.88	4.30

^a^SD is standard deviation,

^b^RSD is relative standard deviation,

^c^Er% is percentage error of the mean.

*LES* lesinurad, *ETC* etoricoxib.

#### Linearity, range and sensitivity

The sample concentrations used to construct the calibration curve were calculated by measuring the peak area ratios of analyte : internal standard between the concentration range 100 to 50,000 ng/mL. The relatively high values of the correlation coefficient (> 0.999) indicated good linear relationships between the analyte concentration and peak area ratios (Table 2). Not to mention the small standard deviation of the intercept (Sa), slope (Sb) and residuals (Sy/x) proving the reliability of the method. Moreover, with a RSD% of the slope (Sb%) less than or equal to 2%, minimal variation between the individual slope values at each point used for establishment of the calibration graph is confirmed (Table 2). The lower limit of quantitation (LLOQ) based on 0.2 mL of rat plasma was 100 ng/mL for the investigated drugs in rat plasma. This concentration limit offers adequate sensitivity to perform a pharmacokinetic investigation to study potential drug-drug interactions between the selected drugs.

#### Stability

Evaluation of the stability of the investigated drugs was performed in plasma and under various conditions (Table 5). Calculation of the concentrations of the drugs in all samples, involved in the stability studies showed satisfactory percentage recoveries (± 10% of their nominal values), Table 5.

**Table 5:** Stability of the studied drugs in rat plasma using the proposed HPLC–DAD method (n = 3).

Stability	LES			EPL			ETC			AMD		
Concentration (ng/ml)	1000	10,000	25,000	1000	10,000	25,000	1000	10,000	25,000	1000	10,000	25,000
Freeze and thaw stability												
Recovery%	91.6	93.8	92.7	91.9	94.3	103.4	98	95.3	96.5	91.3	92.4	95.2
RSD%	1.42	1.53	1.96	2.80	2.18	3.10	5.08	1.67	2.69	5.27	3.61	1.97
Bench-top stability												
Recovery%	92.5	92.5	96.1	105.4	96.8	94.2	97.5	92.8	92.7	93.7	96.3	94.8
RSD%	1.46	1.64	2.28	1.68	1.28	1.47	3.28	2.42	4.16	2.96	3.16	1.78
Long-term stability												
Recovery%	93.8	94.7	94.6	103.5	97.6	92.6	95.8	94.1	90.4	91.3	95.7	92.5
RSD%	2.25	2.34	3.63	1.89	1.75	2.29	1.76	1.92	3.24	4.29	4.27	6.12
Stock solution stability (4^◦^C)												
Recovery%	98.7	97.2	99.2	102.2	97.9	98.9	98.3	96.4	99.2	101.5	99.6	102.1
RSD%	3.37	3.12	1.85	3.74	4.31	1.76	3.63	2.29	3.45	1.57	1.74	1.56
Stock solution stability (room temp.)												
Recovery%	97.3	96.5	94.8	100.7	102.5	102.3	99.5	99.5	98.8	100.5	101.3	101.6
RSD%	1.67	1.27	1.42	1.57	1.64	2.96	1.57	1.73	4.98	3.67	3.24	1.75

*LES* lesinurad, *EPL* eplerenone, *ETC* etoricoxib, *AMD* amiodarone.

#### System suitability testing

The system suitability tests were conducted as per the United States Pharmacopeia (USP)^41^. The calculated parameters were found to be acceptable (Table 3).

## Pharmacokinetic drug–drug interaction study

**Fig. 3:**
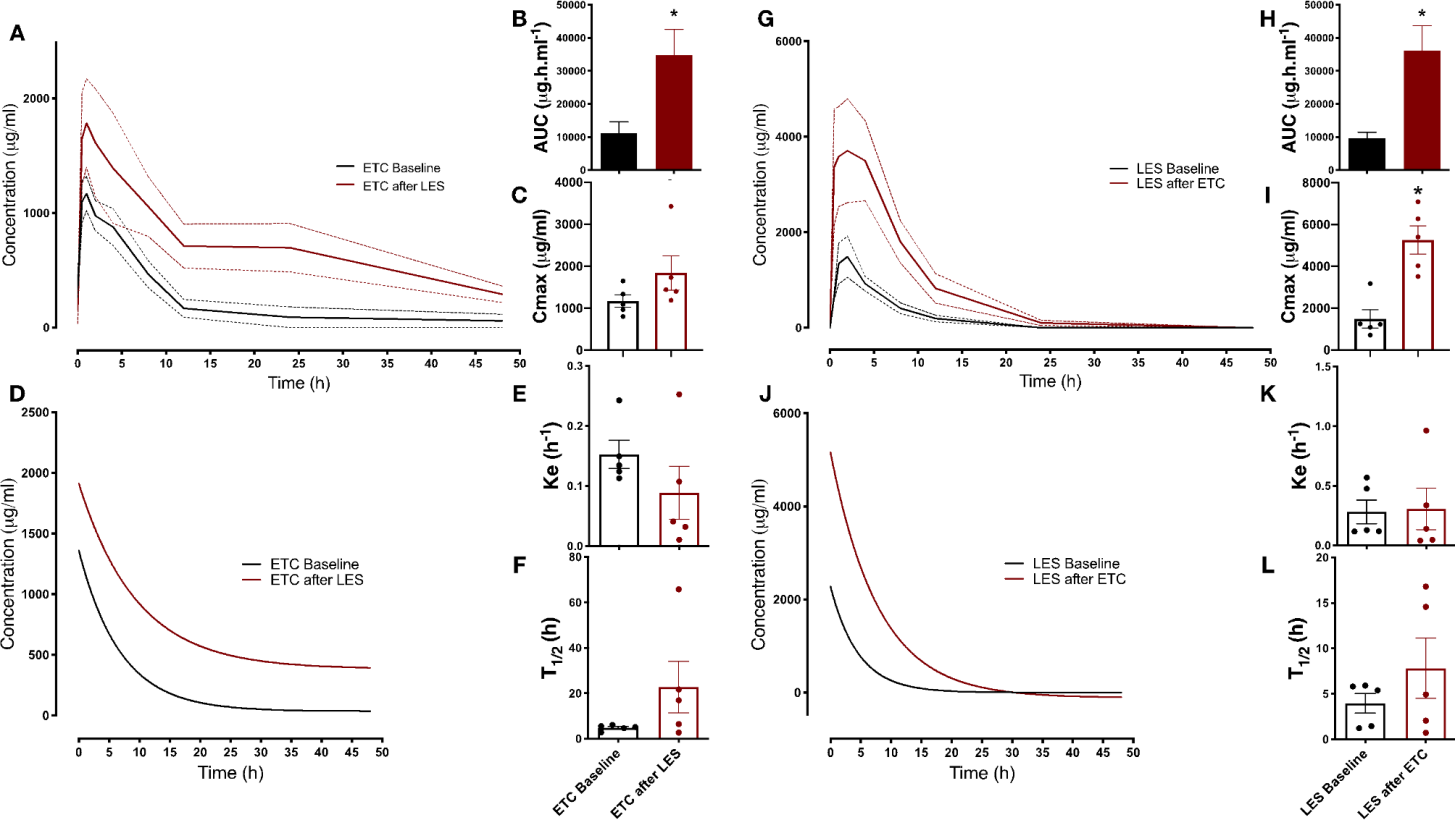
In vivo pharmacokinetics of single oral doses of ETC and LES before and after repeated administration of the other combination component. (**A** & **G**) Plasma concentration vs. time curve of ETC and LES, respectively, alone (black) or after repeated administration of the other component (red). (**B** & **H**) Area under the Plasma concentration vs. time curve (AUC) of ETC and LES, respectively, alone (black) or after repeated administration of the other component (red). (**C** & **I**) Maximal plasma concentration (Cmax) of ETC and LES, respectively, alone (black) or after repeated administration of the other component (red). (**D** & **J**) Best-fit curves for the one-phase decay of plasma concentrations from Cmax to 48 h of ETC and LES, respectively, alone (black) or after repeated administration of the other component (red). (**E** & **K**) Elimination rate constants (Ke) for ETC and LES, respectively, alone (black) or after repeated administration of the other component (red). (**F** & **L**) Elimination half-life (T_1/2_) of ETC and LES, respectively, alone (black) or after repeated administration of the other component (red). Statistical significance for B, C, E, F, H, I, K, and L was determined by Student’s *t*-test. * denotes *P*-value < 0.05.

The results of the assessment of the potential PK interaction between LES and ETC are depicted in Fig. 3 and Table 6. As expected, given the dual impact of either drug on common transporters, the area under the curve (AUC) of each of the two drugs increased following the repeated administration of the other. This effect is likely to be a result of the increased ETC absorption (increased Ka) in the continued presence of LES potentially blocking the intestinal efflux transporters, leading to an increased ETC bioavailability together with decreased tissue uptake as a consequence of transporter inhibition, rather than an alteration of metabolism. Indeed, neither t_1/2_ nor Ke changed for either drug confirming the former conclusion. Certainly, ETC was reported to be subject to metabolism by CYP3A4 ^15^, of which LES is a known inducer^17^, making an increased AUC of ETC as a result of metabolic interaction under these circumstances unlikely. Conversely, it seems that the rate of elimination of either drug was not affected once distribution reached steady state. Significantly, the alteration in distribution appeared to be more serious in LES after repeated ETC administration given the observed sharp increase in Cmax. Such an observation raises a concern of the possible clinical outcome of this interaction both on the therapeutic efficacy of LES potentially not reaching its site of action, or contrarily producing an exaggerated nephrotoxic effect due its increased plasma availability. The latter outcome needs to be also considered in the context of the renal injurious effect of ETC. Finally, there remains the concern that both LES (PMID: 31191704) and ETC (PMID: 18840026) are extensively bound to plasma albumin. However, plasma protein binding for neither LES nor ETC was affected by renal or hepatic impairment (PMID: 31191704, 14517196, 14681341), and no drug interactions due to protein binding displacement were reported, possibly due to the vast excess of plasma albumin content compared to the drug concentration. Additionally, our current method measures the total plasma drug concentration both in the bound and free forms. Nevertheless, there remains a theoretical probability that a combined effect of albumin binding displacement and transporter inhibition could lead to findings similar to those observed in the present study. This can be ascertained in the future with uptake experiments on cell lines expressing known transporters whose findings can be extrapolated and validated by measuring tissue uptake. The developed analytical method could serve as an inexpensive approach to achieve this (Table 6).

**Table 6:** Pharmacokinetic parameters of a single oral dose of ETC and LES alone or after 1 week of repeated oral administration of the other drug.

Drug	AUC (hr.μg/ml)	C_max_ (μg/ml)	T_max_ (hr)	t_1/2_ (hr)	K_e_ (hr^−1^)	K_a_ (hr^−1^)
ETC	11,125 ± 3512	1169 ± 148.3	1	4.878 ± 0.56	0.15 ± 0.02	1.82 ± 0.55
ETC/LES	34,748 ± 7861^a^	1838 ± 406.6	1	22.74 ± 11.3	0.09 ± 0.04	5.18 ± 1.27^a^
LES	9571 ± 1882	1490 ± 433.1	2	3.96 ± 1.07	0.28 ± 0.10	3.32 ± 0.78
LES/ETC	36,146 ± 7558^b^	5263 ± 669.9^b^	2	7.82 ± 3.30	0.31 ± 0.17	3.41 ± 0.45

Data presented are Mean ± SEM.

^a^ and ^b^ denote statistical significance vs. the corresponding value for ETC and LES alone, respectively.

## Conclusion

The multiple co-morbidities commonly diagnosed in gout patients make them at an especially higher risk of drug-drug interactions owing to the simultaneous prescription of multiple drugs. This may affect the clinical course outcomes, potentially precipitating more frequent adverse effects or decreasing drug activity. This work introduces the first simple, and sensitive High Performance Liquid Chromatography (HPLC) method for the simultaneous determination of lesinurad (LES) and other co-administered drugs such as etoricoxib (ETC), eplerenone (EPL), and amiodarone (AMD) in rat plasma. Considering their overlapping clinical indications and pharmacokinetic profiles, these drugs were selected since their co-administration might mutually modify their pharmacokinetic and/or pharmacodynamic consequences. The proposed method was validated according to the FDA bioanalytical guidelines and has exhibited good accuracy and precision. In addition, the method is capable of detecting concentrations as low as 100 ng/mL which empowers it to be utilized as a tool to analyze pharmacokinetic studies samples. Indeed, this method was employed in studying the potential drug-drug interaction between LES and ETC. The pharmacokinetic investigation revealed an increase in the plasma availability of each of the two drugs following the repeated administration of the other. This poses some concerns of the possible reduction in the therapeutic efficacy of LES if it is hindered from reaching its site of action, or on the other hand, the production of an exaggerated nephrotoxic effect. Considering the latter outcome is necessary when co-prescribing LES and ETC regarding the adverse renal effects of ETC. Additionally, this work uncovers the necessity to conduct future pharmacokinetic studies and follow therapeutic dose monitoring for possible adverse effects upon concomitant administration of the selected drugs to gout patients.

## Electronic supplementary material

Below is the link to the electronic supplementary material.

Supplementary Material 1

## Data Availability

Corresponding author can provide the data upon request.
