# Supplementary material for: Simultaneous determination of lesinurad and co-administered drugs used in management of gout comorbidities to uncover potential pharmacokinetic interaction in rat plasma
Source: Sci Rep. 2025 Apr 2;15:11238. doi: 10.1038/s41598-025-93680-4 (PMC11965454; doi:10.1038/s41598-025-93680-4)
Supplement: Supplementary file 1 — Supplementary Material 1 [file 41598_2025_93680_MOESM1_ESM.pdf]

Supporting Information

Simultaneous determination of lesinurad and co-administered drugs used in management of gout comorbidities to uncover potential pharmacokinetic interaction in rat plasma.

Hadeel A. Khalil[1], Amira F. El-Yazbi*[1], Eman I. El-Kimary[1], Mohamed A. Elrewiny[2], Ahmed F. El-Yazbi[2,3], Tarek S. Belal[1].

[1] Pharmaceutical Analytical Chemistry Department, Faculty of Pharmacy, Alexandria University, Alexandria 21521, Egypt

[2]Faculty of Pharmacy and the Research & Innovation Hub, Alamein International University, Alamein 51718, Egypt

[3]Department of Pharmacology and Toxicology, Faculty of Pharmacy, Alexandria University, Alexandria 21521, Egypt

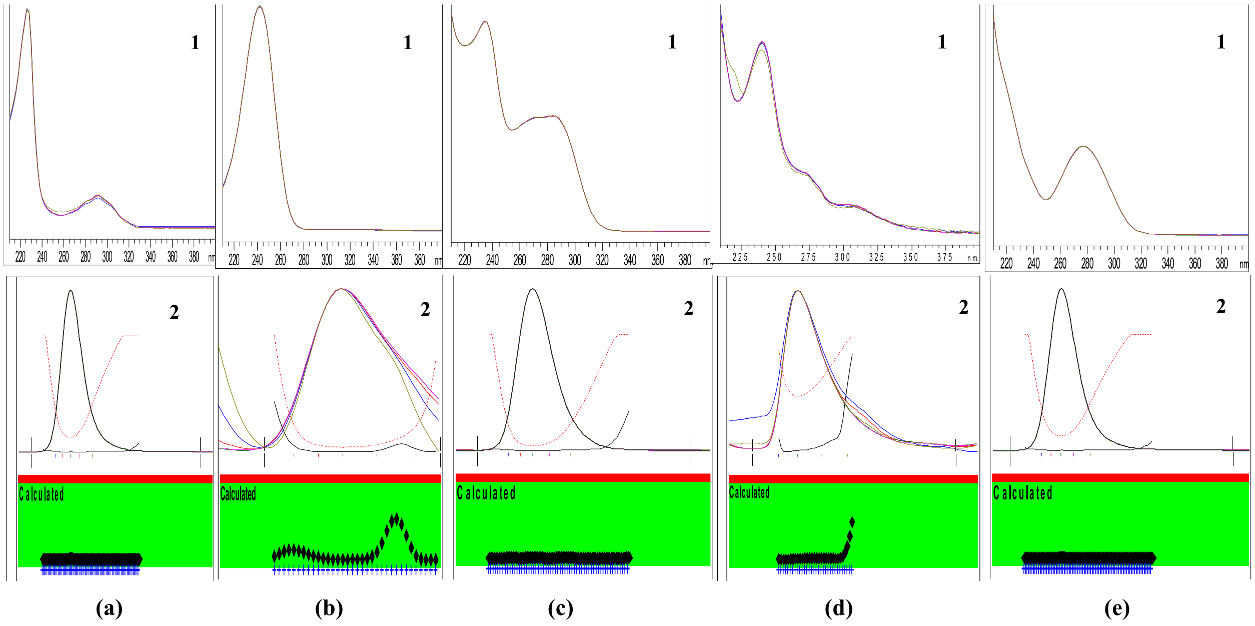

S 1. Superimposed Spectra (1) and similarity curves (2) illustrating peak purity of LES(a), EPL(b), ETC(c), AMD(d) and the internal standard DIC(e) obtained from rat plasma.
